# Outcomes After Proximal Medial Gastrocnemius Recession and Stretching vs Stretching as Treatment of Chronic Plantar Fasciitis at 6-Year Follow-up

**DOI:** 10.1177/10711007231205559

**Published:** 2023-10-30

**Authors:** Martin Okelsrud Riiser, Elisabeth Ellingsen Husebye, Jan Hellesnes, Marius Molund

**Affiliations:** 1 Department of Foot and Ankle Surgery, Department of Orthopaedic Surgery, Østfold Hospital, Grålum, Norway; 2 Department of Foot and Ankle Surgery, Department of Orthopaedic Surgery, Oslo University Hospital, Oslo, Norway; 3 University of Oslo, Faculty of Medicine, Oslo, Norway

**Keywords:** plantar fasciitis, plantar heel pain, PMGR, proximal medial gastrocnemius recession, stretching, long-term outcomes, gastrocnemius tightness, isolated gastrocnemius contracture

## Abstract

**Background:**

Evidence from prospective short-term studies suggest that proximal medial gastrocnemius recession is a safe and efficient procedure to treat chronic plantar fasciitis resistant to nonoperative treatment. The aim of this study was to evaluate the long-term clinical outcomes of proximal medial gastrocnemius recession and stretching compared to a stretching exercise protocol for patients with chronic plantar fasciitis and an isolated gastrocnemius contracture (IGC).

**Methods:**

Forty patients with plantar fasciitis lasting more than 1 year were prospectively randomized to a home stretching exercise program only, or to proximal medial gastrocnemius recession in addition to the stretching program. Clinical and functional data in this study were obtained at baseline and 6-year follow-up. The main outcome was the American Orthopaedic Foot & Ankle Society (AOFAS) ankle-hindfoot score. Secondary outcomes were the visual analog scale (VAS) for pain, the Manchester Oxford Foot Questionnaire (MOxFQ), ankle dorsiflexion, and Achilles complex performance.

**Results:**

Thirty-three of 40 patients completed the 6-year follow-up. Seven patients had crossed over from nonoperative treatment to operative treatment. At 6 years, the operative group demonstrated significantly better outcomes with AOFAS (88.9 vs 78.6, *P* = .012), for pain measured by VAS (2.5 vs 5.5, *P* < .001) and with the MOxFQ total score (24.4 vs 45.9, *P* = .05) (per protocol analysis excluding crossovers). No between-group differences were observed for ankle dorsiflexion or Achilles complex performance at 6 years.

**Conclusion:**

This study demonstrates that the improved function and reduced level of pain by proximal medial gastrocnemius recession and stretching is better compared to stretching alone after 6 years of follow-up for patients with chronic plantar fasciitis and a concomitant isolated gastrocnemius contracture.

**Level of Evidence:**

Level I, randomized controlled trial.

## Introduction

Plantar fasciitis is a painful condition characterized by soreness or tenderness of the plantar surface of the heel.^20^ The prevalence is reported to be 0.5% to 8%.^13,32,37^ One of 10 adult patients will be affected by plantar fasciitis in their lifetime.^29^ In most cases the condition is self-limiting, but 5% to 10% of the patients suffering from plantar fasciitis will not respond to nonoperative treatment and have symptoms exceeding 12 months.^20,27,29,36,38^ The role of isolated gastrocnemius tightness in several foot and ankle pathologies has gained more attention.^11,19,30^ It has been demonstrated that gastrocnemius tightness leads to reduced ankle dorsiflexion, which theoretically, may lead to strain and subsequent pain in the plantar fascia.^26^ There is some evidence that stretching exercises of the gastrocnemius muscle can relieve symptoms.^34^ However, in cases where nonoperative treatment does not resolve symptoms within 12 months, surgery may be considered as a valid treatment option. Traditionally, open plantar fasciotomy has been the surgical treatment of choice. However, studies suggest that this procedure is associated with a long recovery time, a relatively high risk of various complications and low patient satisfaction rates.^7,26,27^ A recent systematic review by Pickin et al^31^ demonstrated that gastrocnemius recession, targeting tightness of the gastrocnemius muscle, is a promising treatment for chronic plantar fasciitis. At present, no long-term studies are published.

We have previously demonstrated that patients with chronic plantar fasciitis and an isolated gastrocnemius contracture (IGC) treated with proximal medial gastrocnemius recession (PMGR) and stretching have less pain and have better function at 1-year follow-up, compared to patients performing stretching alone.^24^ A 6-year follow-up was planned when designing this study. The purpose of the study is to investigate how clinical and functional outcomes may have changed and if the differences in outcomes between groups are maintained after 6 years.

## Material and Methods

This is a randomized controlled trial (RCT). The study was approved by the data protector officer at Oslo University Hospital and the Norwegian National Research Ethics Committees (REK 23380). It is registered at ClinicalTrials.gov (ref. NCT02116478). The inclusion period was between June 2014 and December 2015. The 6-year follow-up was conducted between November 2020 and March 2022. A written informed consent was obtained for every participant.

### Participants

The study included 40 patients aged 18-70 years with plantar fasciitis lasting more than 12 months.

The diagnosis was made based on the clinical symptoms including pain at first step in the morning and pain on palpation of the plantar fascia insertion on the medial plantar aspect of the calcaneus. An isolated contracture of the gastrocnemius (IGC), evaluated by the Silfverskiöld test, had to be present.^5,11,24^ Exclusion criteria were degenerative arthritis of the hindfoot joints or systemic joint disease; previous injury or surgery to the foot or ankle; inability to be informed about the study due to, for example, insufficient language skills; or patient considered inoperable due to comorbidity.

**Figure 1.**
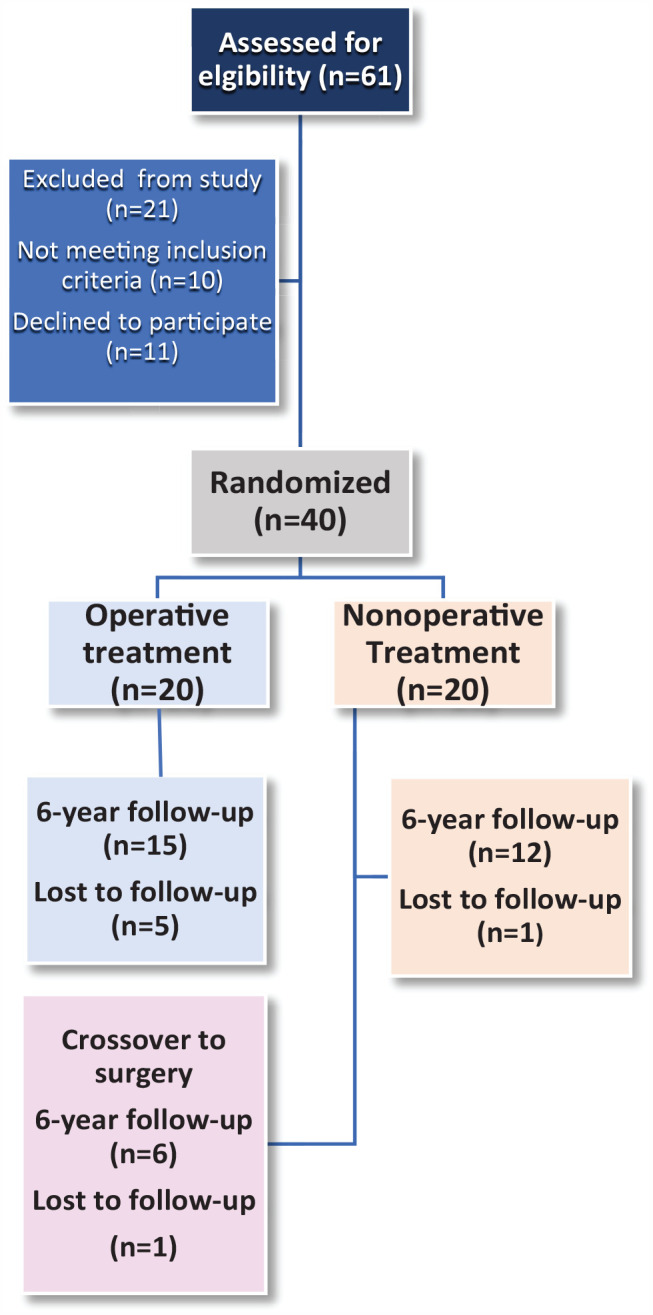
Flow chart describing inclusion and follow-up from baseline to 6 years.

Thirty-three patients met for 6-year follow-up. Seven were lost to follow-up. Of these, 3 did not want to participate in the study and 4 did not respond to follow-up invitation. Seven of 20 nonoperatively treated patients became “crossovers” and were treated with PMGR between the 1- and 6-year follow-up. This left us with 3 patient groups with the following number of patients completing the 6-year follow-up: 15 operatively treated patients, 12 nonoperatively treated patients, and 6 crossover patients (Figure 1).

### Randomization and Anonymizing

After the initial inclusion and after baseline data were obtained, patients were randomized into 2 groups.^24^ At 6-year follow-up, the physical therapist performing biomechanical testing was masked, but the orthopaedic surgeon organizing the follow-up was not.

### Procedures

**Figure 2.**
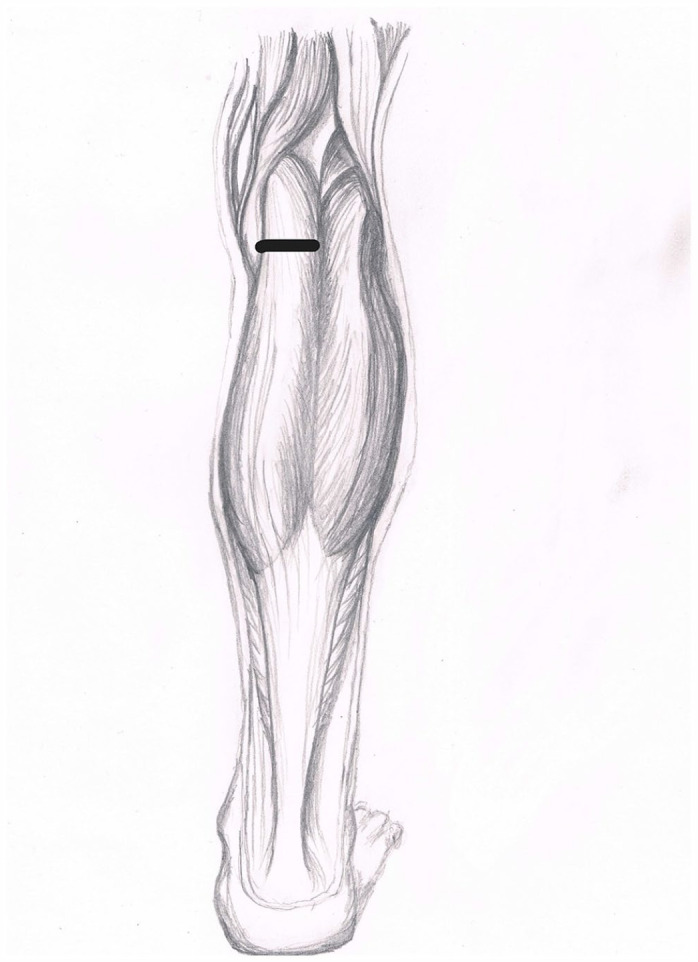
Level of proximal medial gastrocnemius recession. (Courtesy of Maria Serafin)

After primary inclusion, all patients from both groups were instructed by a physical therapist in a standardized stretching exercise home program for 3 months. All patients completed the stretching program, and this was documented through a logbook.^24^ Patients randomized to surgery received a PMGR as described by Barouk.^4^ This procedure involves cutting the tendon sheath of the proximal medial gastrocnemius muscle horizontally and semicircumferentially, through an approximately 4-cm incision, slightly inferior to the popliteal fossa (Figure 2). Patients were instructed to perform the same standardized stretching program directly after they had received surgical treatment. No additional procedures were performed. No control or follow-up on the stretching program was made after the initial 3 months.

### Outcomes

The primary outcome was the American Orthopaedic Foot & Ankle Society (AOFAS) ankle-hindfoot score at 6-year follow-up. This score is a clinician-reported outcome measure constructed by 9 items measuring pain, function, and alignment. The score ranges from 0 to 100, where 100 represents an individual with perfect function, no pain, and no foot/ankle abnormality.

MOxFQ (Manchester Oxford Foot Questionnaire) was added as a secondary outcome measure. MOxFQ consists of 16 questions in 3 domains (pain, walking/standing, and social interaction) leading to a total score from 0 to 100, 0 being the best possible score. It is validated as a reliable tool to measure results of foot and ankle surgery.^8,9^ The 3 domains were originally intended for separate presentation, but the total score has also been demonstrated as a valid parameter.^28^

Visual analog scale (VAS) score for pain at 6 years was another secondary outcome. As plantar fasciitis is associated with a great variance of pain throughout the day, we defined it as “the worst pain you have experienced in your foot within the last 24 hours.” The scale ranges from 0 to 10 points, where 0 represents no pain and 10 the worst imaginable pain.

Ankle dorsiflexion was determined using a device that measured the ankle’s range of motion. It has been previously tested and found to be valid, reliable, and responsive in detecting isolated gastrocnemius contracture (IGC).^25^

Achilles complex (Achilles tendon and gastrocnemius/soleus) performance was evaluated by a test battery consisting of 6 independent tests measured by the Musclelab system (Ergotest Technology, Porsgrunn, Norway). Three tests were jump tests, 2 were strength tests, and 1 was an endurance test.^33^ Nine outcomes from these 6 tests were used for analysis.

### Statistical Analysis

A power analysis was performed before the study. It was based on the assumption that the minimal clinically important difference (MCID) for the main outcome, the AOFAS score, was 10 points. At present, no MCID values exist for AOFAS in this patient group. Ten was considered an adequate difference because several studies have reported MCID values to be in the same range (7.9-30.2, 8.9, 8.9, and 9.5) in treatment of other foot pathologies.^6,10,12,16^ The SD of 10 was estimated based on a similar study on treatment of plantar fasciitis with fasciotomy.^3^ With a power of 80 and level of significance of 5%, 16 patients were needed in each group. Forty patients were included to compensate for possible loss to follow-up. For the 6-year follow-up, statistical analyses were performed using Stata, version 17.0, Standard Edition (StataCorp, College Station, TX).

We used the following treatment allocations to estimate the treatment effect: intention-to-treat (ITT), per protocol (PP), and per protocol excluding crossovers (PPEC). The ITT analysis evaluates treatment effect according to the treatment patients received through baseline randomization, not considering that some of the patients are crossovers. The PP analysis is performed with patients allocated to the treatment received at 6 years. The PPEC analysis is also based on treatment received at 6 years but excluding crossovers to preserve the randomization.

To estimate the treatment effect at 6-year follow-up, we performed a linear mixed effects model for each outcome listed above (except MOxFQ), with random intercept and allocation-time interaction, adjusted for baseline level of the respective outcome. We included all patients with a valid baseline measurement of the respective outcome. Missing data during follow-up are handled implicitly by the model.

The primary effect estimate was defined as the marginal mean difference from the mixed model with PPEC allocation between AOFAS score at 6 years. We chose this allocation because it reflects the treatment patients received at 6 years and it preserves the randomization.

For the ankle dorsiflexion and Achilles complex performance analysis, some patients contributed 2 feet to the analysis. For these data, the model included 2 random intercepts, first the ID of the foot and then the ID of the patient.

Model fit was investigated by looking at the residuals (qnorm plot, scatter plot of fitted vs residuals).

The MOxFQ score was only assessed at the 6-year follow-up (no baseline value). The treatment effect for this outcome was estimated using a linear regression model with the MOxFQ score as dependent and treatment allocation as independent variable.

An external statistician was consulted in determining adequate statistical methods, performing the analyses, and interpreting the results of this study.

## Results

Demographic data such as age, body mass index, duration of symptoms, and sex were comparable between the groups at baseline (Table 1).^24^ No between-group differences in AOFAS or VAS pain were observed at baseline. At 1 year, the AOFAS score and VAS was significantly better in the operated group (Table 1). There were no major complications to surgery. One patient experienced persisted swelling and pain in the popliteal fossa after 1 year and 1 patient experienced prolonged leg cramps, but her heel pain disappeared.^24^ No new complications were registered from 1- to 6-year follow-up.

**Table 1.** Baseline Demographics and Baseline/1-Year Outcomes.^a^

	Operative (n=20)	Nonoperative (n=20)	*P* Value
Age (y)	46 (29-68)	45 (22-63)	.655
Symptom duration (mo)	31 (12-252)	33 (12-396)	.796
Body mass index	27.8 (20.1-49.8)	26.8 (20.2-35.3)	.262
Sex (female/male), n	15:5	16:4	.705
AOFAS score at baseline	59.2 (42-76)	52.5 (37-73)	.357
AOFAS score at 1 y	88 (50-100)	65.5 (31-88)	**<.001**
VAS score at baseline	7.6 (3.9-10)	7.1 (1.5-9.5)	.137
VAS score at 1 y	2.8 (0-8.1)	7.4 (0.2-9.3)	**.001**

Abbreviations: AOFAS, American Orthopaedic Foot & Ankle Society ankle-hindfoot score; VAS, visual analog scale.

a Median values with range are presented unless otherwise noted. Boldface indicates significance (*P* < .05).

The median follow-up time was 76.5 months (range 69-89). Seven patients had crossed over from nonoperative treatment to surgery, and 7 patients were lost to follow-up (Figure 1).

For the main outcome, AOFAS ankle-hindfoot score, there were significantly better results in the group that received operative treatment and stretching (operative group) vs the group that received stretching alone (nonoperative group) at 6 years. These findings were consistent in the ITT, PP, and PPEC analysis (Table 2). Using the same models, significantly better VAS scores were also found for the operative group at 6 years (Table 2).

**Table 2.** AOFAS Ankle-Hindfoot Score and VAS at 6-Year Follow-up.^a^

	Operative, Marginal Mean (95% CI)	Nonoperative, Marginal Mean (95% CI)	Marginal Mean Difference (95% CI)	*P* Value
AOFAS ankle-hindfoot score: 6 y				
ITT	88.8 (83.2, 94.2)	79.2 (74.0, 84.4)	9.5 (2.0, 17.1)	.013
PP	86.6 (81.9, 91.3)	78.8 (72.8, 84.9)	7.8 (0.3, 15.3)	.042
PPEC	88.9 (83.6, 94.2)	78.6 (72.8, 84.5)	10.2 (2.3, 18.2)	.012
VAS score: 6 y				
ITT	2.5 (1.3, 3.6)	5.6 (4.6, 6.6)	−3.1 (–4.6, –1.6)	<.001
PP	3.5 (2.5, 4.4)	5.3 (4.1, 6.6)	−1.9 (–3.4, –0.3)	.019
PPEC	2.5 (1.4, 3.6)	5.5 (4.3, 6.7)	−3.0 (–4.6, –1.4)	<.001

Abbreviations: AOFAS, American Orthopaedic Foot & Ankle Society; ITT, intention to treat; PP, per protocol; PPEC, per protocol excluding crossovers; VAS, visual analog scale.

a Number of patients in the analysis: Operative: ITT = 20, PP = 27, PPEC = 20. Nonoperative: ITT = 20, PP = 13, PPEC = 13.

Linear regression analysis, grouping patients by ITT, demonstrated better MOxFQ scores in the operative group for the total score and all the subscores. For the PPEC analysis, only the total score showed significant differences. Remaining PPEC results and all PP results pointed in the same direction but were not significant (Table 3). The exact number of patients in each group in all ITT, PP, and PPEC analyses are listed in Tables 2 and 3.

**Table 3.** Manchester Oxford Foot Questionnaire at 6-Year Follow-up.^a^

	Operative, Mean (SD)	Nonoperative, Mean (SD)	β Coefficient (95% CI)^b^	*P* Value
MOxFQ: total score				
ITT	24.4 (6.8)	50.5 (6.2)	−26.0 (–44.7, –7.4)	.008
PP	34.4 (6.3)	45.9 (8.3)	−11.5 (–32.8, 9.8)	.280
PPEC	24.4 (7.0)	45.9 (7.8)	−21.5 (–43.0, 0.00)	.05
MOxFQ: walking/standing				
ITT	26.4 (7.4)	57.8 (6.7)	−31.4 (–51.8, –11.1)	.004
PP	37.2 (6.9)	54.6 (9.1)	−17.3 (–40.7, 6.0)	.140
PPEC	26.4 (7.6)	54.6 (8.5)	−28.2 (–51.6, –4.7)	.02
MOxFQ: pain				
ITT	28.7 (7.5)	52.2 (6.8)	−23.6 (–44.2, –2.9)	.027
PP	38.1 (6.8)	47.5 (9.0)	−9.4 (–32.3, 13.5)	.409
PPEC	28.7 (7.9)	47.5 (8.8)	−18.8 (–43.1, 5.5)	.12
MOxFQ: social				
ITT	15.7 (5.7)	33.9 (5.2)	−18.2 (–33.9, –2.5)	.025
PP	24.4 (5.2)	27.8 (6.9)	−3.4 (–21.0, 14.2)	.696
PPEC	15.7 (5.5)	27.8 (6.2)	−12.1 (–29.1, 4.9)	.156

Abbreviations: ITT, intention to treat; MOxFQ, Manchester Oxford Foot Questionnaire; PP, per protocol; PPEC, per protocol excluding crossovers.

a Number of patients in the analysis: Operative: ITT = 20, PP = 27, PPEC = 20. Nonoperative: ITT = 20, PP = 13, PPEC = 13.

b Beta coefficient showing the mean difference comparing the surgery group to the control group (reference).

Ankle movement, measured by ankle dorsiflexion, showed no differences between the groups when measured with straight knee or with the knee flexed (Table 4). Only the PP analysis is presented in Table 4. No significant differences were found in ITT or PPEC analysis.

**Table 4.** Ankle Dorsiflexion and Achilles Complex Test Battery: 6 Years.

Outcome	Operative, Marginal Mean (95% CI)	Nonoperative, Marginal Mean (95% CI)	Marginal Mean Difference (95% CI)	*P* Value
Ankle dorsiflexion, degrees				
Straight knee	7.6 (6.1, 9.0)	6.7 (4.9, 8.6)	0.8 (–1.6, 3.3)	.499
Flexed knee	23.4 (21.3, 25.6)	23.2 (20.5, 26.0)	0.2 (–3.2, 3.7)	.898
Hopping, jumps/second	1.7 (1.6, 1.9)	1.4 (1.2, 1.6)	0.3 (0.1, 0.6)	.006
VCMJ max height, cm	4.4 (3.8, 4.9)	5.0 (5.7, 4.4)	−0.7 (–1.5, 0.1)	.109
DCMJ max height, cm	4.5 (3.9, 5.2)	3.9 (3.1, 4.8)	0.6 (–0.4, 1.6)	.258
CTR resistance, W				
23 kg	198 (149, 246)	228 (168, 287)	−30 (–105, 45)	.434
33 kg	212 (165, 260)	226 (161, 292)	−13 (–92,65)	.731
ECTR resistance, W				
23 kg	185 (152, 217)	274 (232, 316)	−89 (–144, –34)	.001
33 kg	183 (150, 217)	242 (194, 291)	−59 (–119, 1)	.053
Number of toe raises	20 (17, 24)	22 (18, 26)	−2 (–7, 3)	.376
Toe raises total work, J	1184 (971, 1398)	1340 (1085, 1595)	−155 (–489, 178)	.362
Number of feet (PP)^a^	39	20		

Abbreviations: CTR, concentric toe raise; DCMJ, drop counter movement jump; ECTR, eccentric concentric toe raise; VCMJ, vertical counter movement jump.

a Results are analysed PP (per protocol).

The Achilles complex test battery found differences between the groups in 2 of the 9 outcomes (Table 4). According to these tests, the operative group had a higher jumping frequency, and the nonoperative group had a higher power performance on one of the 4 strength outcomes. The ITT and PPEC analysis did not reveal any other differences than those described for the PP analysis.

## Discussion

The study demonstrates improved long-term function and reduced level of pain by PMGR surgery and stretching compared with stretching alone for patients with chronic plantar fasciitis in combination with an isolated gastrocnemius contracture. The effect of surgery is not reduced after 6 years. A long-term difference in ankle dorsiflexion could not be found between the groups, and no clinically relevant differences in performance of the Achilles complex could be demonstrated.

Molund et al^24^ demonstrated that PMGR surgery is a safe and efficient procedure to treat chronic plantar fasciitis in patients with an isolated gastrocnemius contracture. Other studies have demonstrated similar findings.^1,14,15,18,23,27^ However, the maximal follow-up time of any prospective study is 12 months. The findings of this study, with a 6-year follow-up, are therefore unique and give important knowledge to clinicians treating patients with chronic plantar fasciitis. When treating chronic plantar fasciitis with PMGR, good long-term results can be expected.

The main strength of this study is that it is a randomized controlled trial, and that the follow-up time is long. The follow-up rate of 82.5 percent is good considering that 6 years has passed since primary inclusion. Another strength is that patients are evaluated by several clinical outcomes.

The main limitation of the study is its small study population. More robust results would have been achieved in a larger study. Seven patients were lost to follow-up, and most missing patients were found in the group primarily allocated to surgical intervention. By doing a linear mixed model analysis on our results, the loss to follow-up is handled. When designing the study, a power analysis was made that required 16 patients in each group. The PP and PPEC analysis can therefore be considered underpowered.

Another limitation of the study is the quality of the primary outcome: the AOFAS ankle-hindfoot score. This tool is widely used and gives clinicians the opportunity to compare results of many studies. However, the AOFAS ankle-hindfoot score has been criticized for its lack of responsiveness, poor inter- and intrarater reliability, ceiling effects, and for its nature of being a clinician-reported outcome, where the opinion of the examining clinician heavily affects the result.^17,22,35^ In future studies, we will refrain from using the AOFAS ankle-hindfoot score as a primary outcome. In this setting, we had no choice but to continue using the outcomes that were already given. To provide support to our conclusions, we added the MOxFQ, which also can capture the patient’s perspective. The visual analog scale also supports the primary outcome results.

The statistical analysis of our data was complicated by 7 missing patients and 7 patients crossing over from nonoperative to operative treatment after the 1-year follow-up. By running the linear mixed effects model allocating patients to ITT, PP, and PPEC we could add sensitivity to our result and compensate for missing data. For the main outcome AOFAS score and for VAS for pain, all analysis pointed in the same direction and showed significant differences between the groups. For the primary effect estimate, which was the marginal mean difference from the mixed model with PPEC allocation between AOFAS score at 6 years, the difference surpassed what was set as minimally clinical important difference (MCID) of 10 points. However, no studies have estimated the MCID for the AOFAS score in this patient group. MCID values from studies on other foot pathologies had to be considered.^6,8,12,16^ Compared to these values, the results of the main outcome of this study can be considered clinically significant. The same conclusion can be drawn for the VAS pain results that surpassed the estimated MCID from a study on plantar heel pain by Landorf et al^21^ (0.8-1.9).

For the secondary outcome, MOxFQ, there were differences in favor of surgery by all methods of analysis, for both the total and the subdomains. However, only the ITT and PPEC analysis could demonstrate significant differences. The MOxFQ results support the main conclusion, but because no baseline data exist for the MOxFQ, this result should only be interpreted in an exploratory or descriptive manner. In this regard, it can be noted that in the walking/standing and in 2 of the pain subdomain analyses (ITT and PPEC), the MOxFQ reached the threshold of the MCID values of 16 and 12.^8^ In the PP analysis for the pain subdomain and for all the social interaction analyses the differences between the groups did not surpass the MCID.

When descriptively comparing 6-year results to baseline, a greater increase in the AOFAS ankle-hindfoot score and greater decrease in VAS is seen in the operative group (Tables 1 and 2). When comparing with the 1-year results (Table 1), we see that the AOFAS score for the operative group has remained at the same level (88.0 vs 88.6-88.9), but the AOFAS score for the nonoperative group has increased from 1 year to 6 years (65.5 vs 78.8-79.2). The improvement of the AOFAS score in the nonoperative group can probably be explained by the effect of crossovers. The patients crossing over to surgery after 1 year are the patients with the lowest AOFAS scores. When crossing over to surgery, the remaining (and most satisfied) patients increase the overall score of the nonoperative group.

From baseline to 6 years, no clinically relevant changes in ankle dorsiflexion could be seen in patients receiving surgery (6 degrees [−3, 15] vs 7.6 degrees [6.1, 9.0]). The increase in ankle dorsiflexion seen between baseline and 1 year (6 vs 10.5 degrees)^24^ could not be reproduced. Further, no difference in ankle dorsiflexion could be demonstrated between the operative and nonoperative group at 6 years. The postulated effect mechanism of PMGR is that reducing IGC will secondarily provide less strain on the plantar fascia.^26^ Because patients in the operative group experienced less pain and better function than the nonoperative group, one could also expect the difference in ankle dorsiflexion to be greater. Our data do not provide enough information to give any conclusions to this question. One hypothesis is that the difference between the groups exists, but that our measuring methods and the small size of the study population, prevents us from demonstrating this. Another hypothesis is that a transient elongation of the gastrocnemius muscle is enough to relieve plantar fasciitis symptoms. These hypotheses cannot be verified or discarded by the results of this study. More studies are needed to observe the long-term effects of PMGR on ankle dorsiflexion.

The Achilles complex test battery evaluated different modalities in Achilles complex performance. At 6 years, our data showed minor differences between the groups that cannot be considered clinically relevant. Our data are too small to make any certain conclusions on this matter. However, and most importantly, the results indicate that no obvious negative effect on Achilles complex performance can be seen in patients treated with PMGR. Hypothetically, cutting the tight outer layer of the medial gastrocnemius muscle may reduce the function of the muscle, but no such effect can be demonstrated.

This study finds no functional reduction and a low rate of complications,^24^ which suggest that that PMGR can be considered a safe procedure. Pickin et al^31^ present similar findings in a systematic review of 7 different studies on PMGR as treatment for chronic plantar fasciitis. The few complications that arose were mainly related to infection or to irritation/damage to the sural nerve. Sural nerve complications had all resolved within 1 year.

Chronic plantar fasciitis may be mimicked by other foot pathologies.^2^ No recognized standard for diagnosing the condition exists. In this study, clinical signs were used as diagnostic tools to include patients. Neither magnetic resonance imaging nor ultrasonography were used routinely to diagnose patients. To our knowledge, no imaging can provide a gold standard for diagnosing plantar fasciitis, but when in doubt, they may be a useful guide. Because of randomization, these potentially “mis-diagnosed” patients should be distributed equally in the 2 groups. In our material, we can see that some patients, who received surgery, had no clinical effect of the procedure. This may indicate that diagnosing plantar fasciitis and IGC is complicated and that diagnostic criteria could have been more rigid in our study. It may also indicate that it might be additional contributing factors than IGC that has a role in the development of chronic plantar fasciitis.

## Conclusion

After 6 years, patients with chronic plantar fasciitis in combination with an isolated gastrocnemius contracture treated with proximal medial gastrocnemius recession and stretching have less pain and better function than patients treated with stretching alone. No differences in Achilles complex performance can be seen between the intervention and the control group. This supports the conclusion that PMGR is a safe procedure, with good long-term results, that can be recommended to patients with chronic plantar fasciitis with a concomitant gastrocnemius contracture resistant to nonoperative treatment.

## Supplemental Material

sj-pdf-1-fai-10.1177_10711007231205559 – Supplemental material for Outcomes After Proximal Medial Gastrocnemius Recession and Stretching vs Stretching as Treatment of Chronic Plantar Fasciitis at 6-Year Follow-up Click here for additional data file. Supplemental material, sj-pdf-1-fai-10.1177_10711007231205559 for Outcomes After Proximal Medial Gastrocnemius Recession and Stretching vs Stretching as Treatment of Chronic Plantar Fasciitis at 6-Year Follow-up by Martin Okelsrud Riiser, Elisabeth Ellingsen Husebye, Jan Hellesnes and Marius Molund in Foot & Ankle International
